# Comparison between Two Different Two-Stage Transperineal Approaches to Treat Urethral Strictures or Bladder Neck Contracture Associated with Severe Urinary Incontinence that Occurred after Pelvic Surgery: Report of Our Experience

**DOI:** 10.1155/2012/481943

**Published:** 2012-04-24

**Authors:** A. Simonato, M. Ennas, A. Benelli, A. Gregori, F. Oneto, E. Daglio, P. Traverso, G. Carmignani

**Affiliations:** ^1^Clinica Urologica “L. Giuliani”, DISC, IRCCS Azienda Ospedaliera Universitaria San Martino-IST, Genoa, Italy; ^2^Department of Urology, Ospedale “L. Sacco”, Milan, Italy

## Abstract

*Introduction.* The recurrence of urethral/bladder neck stricture after multiple endoscopic procedures is a rare complication that can follow prostatic surgery and its treatment is still controversial. *Material and Methods.* We retrospectively analyzed our data on 17 patients, operated between September 2001 and January 2010, who presented severe urinary incontinence and urethral/bladder neck stricture after prostatic surgery and failure of at least four conservative endoscopic treatments. Six patients underwent a transperineal urethrovesical anastomosis and 11 patients a combined transperineal suprapubical (endoscopic) urethrovesical anastomosis. After six months the patients that presented complete incontinence and no urethral stricture underwent the implantation of an artificial urethral sphincter (AUS). *Results.* After six months 16 patients were completely incontinent and presented a patent, stable lumen, so that they underwent an AUS implantation. With a mean followup of 50.5 months, 14 patients are perfectly continent with no postvoid residual urine. *Conclusions.* Two-stage procedures are safe techniques to treat these challenging cases. In our opinion, these cases could be managed with a transperineal approach in patients who present a perfect operative field; on the contrary, in more difficult cases, it would be preferable to use the other technique, with a combined transperineal suprapubical access, to perform a pull-through procedure.

## 1. Introduction

Referring to prostate surgery, urinary incontinence and iatrogenic bladder neck/urethral strictures are devastating complications that strongly impair a patient's quality of life (QoL).

Iatrogenic late urinary incontinence following surgery for benign prostatic hyperplasia (BPH) is an uncommon complication occurring in less than 1% of cases, especially because of technical improvements during the last decade in performing a transurethral resection of the prostate (TURP) [1–3].

In a recent review of more than 50 papers, the weighted mean continence rate at 12 months after retropubic radical prostatectomy (RRP), laparoscopic radical prostatectomy (LRP) and robot assisted radical prostatectomy (RARP) was 79%, 84.8% and 92% respectively. However, persistent post-RP urinary incontinence in literature affects 2% to 5% of patients one year after surgery [4, 5]. This broad range of incidence is obviously influenced by different factors such as the surgeon's experience, surgical technique, selection of the patients and time of assessment relative to surgery [6].

Urethral strictures are the major late complication after TURP, ranging from 2.2% to 9.2% [6]; the range is from 0.6% to 14% considering also open simple prostatectomies (OSP) for BPH [7].

Anastomotic strictures following radical prostatectomy for prostate cancer are reported in about 1–8% of all patients [8].

Most of the strictures are managed with dilatation or endoscopic treatments. Eventual severe incontinence after incision of the stricture can be successfully managed by implantation of an artificial urinary sphincter (AUS) [9]. The treatment of strictures in incontinent patients after failure of transurethral procedures is controversial: a permanent stent or a urinary diversion (by catheters or major surgery) does not always achieve an optimal functional result, which is the combination of lumen patency and urinary continence [10]. Some authors advocate complex abdomino-perineal approaches to perform urethroplasty and AUS implantation in one or two stages [3, 11], whereas others perform a one- or two-stage implantation of prostatic stent and AUS [10, 12]. However, such procedures are complex, invasive, and potentially morbid.

In this paper we report our experience in the management of patients with combined urinary incontinence and urethral/bladder neck stricture after prostatic surgery; the surgical treatment of these patients is continually evolving and we made our choices and evaluations considering the individual patients, the surgeon's experience, and the available resources.

We approached the patients with two different two-step techniques; an open urethroplasty followed by AUS insertion after 7 months and an urethroplasty with a pull-through technique followed by AUS insertion after 8–10 months.

## 2. Materials and Methods

### 2.1. Patients

We retrospectively evaluated 17 patients, treated at our institution between September 2001 and January 2010 for a combination of severe urinary incontinence and posterior urethral stricture or bladder neck contracture after prostate surgery. Fourteen patients had an anastomotic bladder neck contracture following RP for localized prostate cancer. The other 3 patients developed a posterior urethral stricture after prostatic surgery for BPH: 1 after TURP and 2 after OSP. Among these last 3 patients, 2 presented a type II prostatic urethral stricture and 1 a type III stricture according to the criteria of Pansadoro and Emiliozzi [7].

All the patients presented with erectile dysfunction. Two patients underwent adjuvant radiotherapy after RP, 2 patients suffered from diabetes mellitus and 1 from chronic hepatitis C. Before definitive treatment, all the patients underwent 4 or more internal urethrotomies or transurethral resections (Table 1) followed by recurrence of disease.

To exclude detrusor over-activity or compliance abnormalities, every patient was evaluated through physical examination and a diagnostic work-up including flexible urethroscopy, retrograde and voiding urethrogram, and urodynamic investigations, according to the methodology and definitions of the International Continence Society.

The patients were scheduled for a two-step approach: first they underwent urethroplasty and subsequently, the implantation of an AUS. Retrospectively we identified two subgroups: in the first one (group A) 6 patients underwent an anastomotic urethroplasty with removal of scar tissue and repetition of end to end anastomosis (13). In the second one (group B) 11 patients were subjected to urethroplasty with a pull-through technique following the Solovov-Badenoch principle. In group A the AUS were implanted after a follow up period of 6 months and in group B after 8–10 months.

### 2.2. Surgical Technique: The First Step

While the patient is on call to the operating room (OR), antibiotic intravenous prophylaxis is administered and the hair is removed from the surgical field in the OR just prior to surgery.

All the procedures are performed with the patients in the lithotomy position.

#### 2.2.1. Trans-Perineal Urethroplasty with End to End re-Anastomosis

During a flexible urethroscopy a guide-wire is passed into the bladder. Then a reversed Y-shaped incision is made on the perineal skin; the layers below are opened, up to the bulbospongiosus muscles, which are separated in order to expose the bulbar urethra. After that a vascular loop is passed around the bulbar urethra.

A 24 Ch Catheter is passed into the urethra to recognize the distal edge of the stricture, so that we are able to remove dorsal scarring tissue of the stricture from the urethral lumen to the periphery, until healthy tissue is observed.

**Figure 1:**
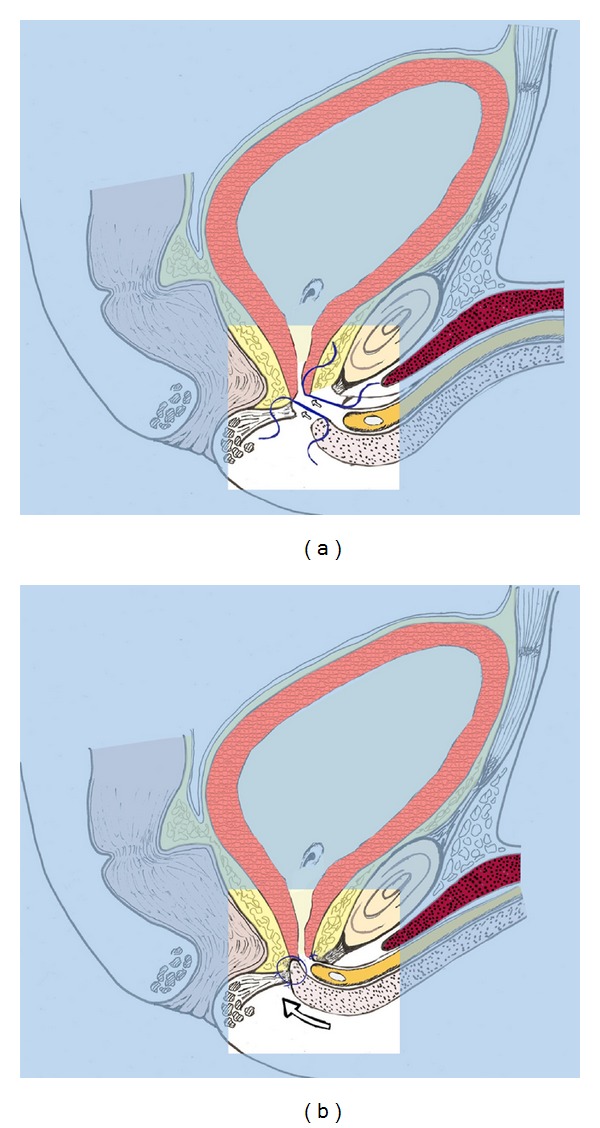
(a) Two 3–0 polygalactinin sutures are passed through the proximal edge of the urethra and the bladder neck. (b) The sutures are tied.

To obtain a tension-free anastomosis, the anterior urethra is largely dissected from the corporal bodies and the intercrural space is developed, starting from the bifurcation of the corporal bodies, with a wide mobilization. After that, we dorsally spatulate the anterior urethra and interrupted 3–0 polygalactin acid sutures are placed on the proximal mucosal edges; sutures are then placed in the distal segment of the urethra (Figure 1(a)) and tied after placement of a 18 Ch catheter (Figure 1(b)). Furthermore, four more interrupted sutures are placed between urethra and corporal bodies to better guarantee a tension-free anastomosis.

Finally, a non-absorbable suture is passed between urethra and corporal bodies as a landmark to identify the correct place to place the cuff at implantantion of the AUS.

The incision is closed in layers, after which the bulbourethral muscles are reconstructed and the superficial perineal fascia is also closed.

A fourteen-day course of antibiotics is given and the catheter is removed at day 10 after a cystography.

#### 2.2.2. Repetition of the Vesico-Urethral Anastomosis with the Pull through Technique

The procedure starts with a perineal reversed Y shaped incision and exposure of the bulbar urethra, separating the bulbospongiosus muscles; a vascular loop is then passed around the bulbar urethra. The distal edge of the stricture is recognized with the help of a Nelaton urethral sound and then incised.

The bladder is then punctured suprapubically with a needle, a guide-wire is passed through and the tract is coaxially dilated until a 26 Ch Amplatz sheath is placed; through this, a flexible cystoscope is introduced into the bladder and a guide-wire is passed through the bladder neck to the stenosis and retrieved from the perineum. A 24 Ch Nelaton urethral sound is subsequently suprapubically passed through the guide-wire and introduced up to the bladder neck.

The dorsal scarring tissue of the stricture is proximally removed by pulling and following the Nelaton catheter, with whom the guide-wire moves in unison, until healthy tissue is observed (to create a large lumen for the vesico-urethral anastomosis). The anterior urethra is largely dissected from corporal bodies and the intercrural space is developed with a wide mobilization in order to obtain a tension-free anastomosis. The anterior urethra is then spatulated dorsally and interrupted polygalactin acid 3-0 sutures are placed on the proximal edges of the corpus spongiosus of the urethra to guarantee haemostasis.

**Figure 2:**
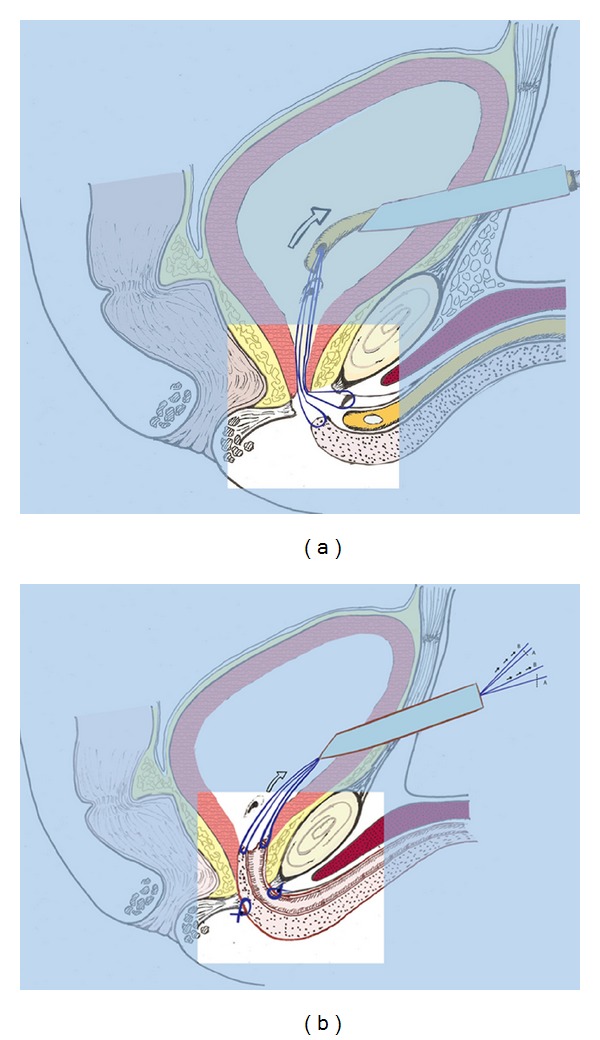
(a) Placing of 2 0-0 sutures at the proximal edge of the anterior urethra. They pass into the Nelaton supra-pubical catheter. (b) The proximal edge of the urethra is pulled through the pelvic floor and placed inside the Bladder. Polygalactinin 3–0 sutures are then placed in the proximal segment of the urethra between the para-urethral fascia and the vesico-urethral anastomosis. Afterwards the sutures exiting the Amplatz catheter are cut (A) and pulled out (B) of the bladder.

Two mono-filament 0-0 sutures are placed at the proximal edge of the anterior urethra; afterwards they are passed through the Nelaton catheter from its tip until the proximal edge of the sound (Figure 2(a)). Therefore this is then carefully retrieved through the perineum into the bladder neck to pull the proximal stump of the urethra inside the bladder through the bladder neck. A gentle trans-perineal push of the urethra with the fingers helps to successfully complete the manoeuvre, so that we can define the procedure as a combined “pull through and push through technique”. Finally, the sutures exiting the Amplatz catheter are cut and pulled out of the bladder.

After placement of a 18 Ch catheter, interrupted polygalactin 3-0 sutures are then placed in the proximal segment of the urethra between the paraurethral fascia and the vesico-urethral anastomosis to achieve a watertight anastomosis (Figure 2(b)). At this point the urethra is evaluated to find the suitable place to place the cuff of the AUS during the second surgery and a monofilament non-absorbable suture is passed as a future landmark. Four more sutures are placed between the corpus spongiosus of the urethra and the corporal bodies in order to obtain a better tension-free anastomosis; this manoeuvre is accomplished carefully to avoid the placement of the stitches where the cuff will be placed.

The bulbo-urethral muscles are reconstructed and the superficial perineal fascia is re-established. The incision is then closed in layers.

The two mono-filament 0-0 sutures placed at the proximal edge of the urethra are removed pulling a distal tail, after which a suprapubic 14 Ch catheter is placed.

A fourteen-day antibiotic course is given and ten days later, surgery patients undergo cystography in order to remove the urethral catheter; the suprapubical catheter is extracted three days later.

### 2.3. Follow up after the First Step

All patients were evaluated with urine cultures one, three and six months later. After six months a flexible urethroscopy was also performed.

If a stable patent urethral lumen was present and the patient was completely incontinent, he was scheduled for AUS placement (7 months after the first surgery in group A and 8 to 10 months after surgery in group B).

### 2.4. Surgical Technique: The Second Step, Trans-Perineal AUS Insertion

While the patient is on call to the operating room (OR), antibiotic intravenous prophylaxis (gentamicin sulphate plus vancomycin) is administered. The hair is removed from the surgical field in the OR just prior to surgery.

The elements of the system are immersed into an antibiotic solution.

The patient is placed in the lithotomy position and a vertical midline perineal incision is made. Then the landmark suture placed during the previous surgery is located; it is useful to find the plane between the urethra and the corporal bodies. The urethra is circumferentially dissected off the corporal bodies for a length of 2 cm to accommodate the cuff of the sphincter. The circumference of the urethra is then measured for cuff size selection.

Afterwards, a small incision in the right iliac region is made, and a pocket bluntly created under the rectus muscle, extra-peritoneally, to allow the placement of the balloon reservoir. The reservoir tubing is brought out through a separate incision in the anterior rectus fascia to avoid scrotal violations.

The cuff tubing is grasped, and guided up into the abdominal wound passing through the bulbo-urethral muscles. Then a lateral subcutaneous hemi-scrotal pouch is created with Hegar dilators, to place the pump of the AUS. Before its placement the pump is carefully filled with saline solution.

All of the appropriate tubing connections are made and the device is tested and deactivated. The cuff placed around the bulbar urethra is 4-5 cm in length and a 61 to 70 cm H20 pressure-regulating balloon is used. Finally, the incisions are closed in layers.

A fourteen-day course of antibacterial therapy is given and the devices are activated 4 weeks after surgery.

### 2.5. Follow up after the AUS Insertion

Every three months for the first year and then annually, we carried out an examination and evaluated urine cultures and post-void residual urine.

## 3. Results

We did not observe intraoperative or early postoperative complications in either of the approaches, for any patient. Six months after the first step of the treatment 16 patients (94%) were completely incontinent with no urethral strictures and complete anastomotic healing. One patient was retentive after the urethroplasty but he showed a pervious urethra lumen and continues to drain his bladder with self-catheterization.

All 16 patients underwent AUS implantation; 10 of them (58.8%), from group B, after an eight/ten-month follow up and 6 of them (35.3%), the entire group A, after a six-month follow up.

After a mean follow up of 50.5 months (range 18–111) 14 patients (82.4%) are continent without post-void residual urine and a perfectly functional device.

Only two of the 16 patients who had undergone AUS implantation needed a complete removal of the device due to urethral erosion. One of them was a previously irradiated patient who developed urethral erosion 6 months after AUS implantation; the other one was a patient with chronic hepatitis C who presented scrotal swelling and partial urethral erosion 2 months after device implantation; the former was part of group A and the latter of group B. However both of them are now completely incontinent with a pervious urethral lumen.

Therefore we achieved an overall success rate of 5/6 (83.3%) in group A and of 9/11 (81.8%) in group B.

The patient presenting chronic hepatitis underwent scrotal and abdominal ultrasonography which showed a low level of echogenicity around the pump and the balloon. The microbiological examination of the liquid obtained by the fine-needle aspiration revealed no bacterial contamination.

## 4. Discussion

For both the anastomotic posterior urethroplasty techniques we achieved excellent results, with a specific success rate of 100% in group A and 91% in group B, similar to contemporary reported experiences concerning the trans-perineal approach [14]. Regarding AUS implantation, currently it seems to be the most effective treatment for severe urinary incontinence [15]. Having said this, in our opinion, in patients affected by recurrent urethral stricture caused by prostatic surgery, it is a reasonable approach to perform preliminary surgery to obtain a patent and stable lumen. The aim of this first step is to create a clinical and functional context of urinary incontinence which can be managed by the implantation of an AUS.

We believe that the first step of treatment would have to be a trans-perineal or combined trans-perineal-supra-pubic (endoscopic) access; these approaches are less invasive and have a lower rate of perioperative morbidity than the open supra-pubic techniques [16, 17] as demonstrated by the absence of early post-operative complications in our series. Concerning the selection of surgical techniques to be performed as the first step of the procedure, we can explain the choices given by considering different anatomical and clinical aspects. End to end anastomosis is easy to perform when the bladder neck is joinable without particular problems due to favourable local conditions, that is, when the angle between the ischio-pubic ramus is wide and the scarring tissue is poorly or moderately represented. On the contrary, when we encounter patients with acute angles between the ischio-pubic ramus or with too much fibrous tissue, we prefer to adopt the pull-through technique which plays on the Solovov-Badenoch principle [18, 19]. This technique was abandoned because of the low success rate, nevertheless we believe that those poor results were due to adverse operative and anatomical conditions: originally this approach was intended for the treatment of posterior urethral traumatic injuries, but repair of an unaligned urethra in these patients may be very difficult owing to extremely distant urethral edges (e.g., dislocated by a pelvic hematoma)and the addition of bone scarps in the surgical field. In our series, a wide dissection of the corpus spongiosus from the corpora cavernosa always allowed adequate mobilization of the distal urethra so that the surgeon encountered no problems in placing the proximal edge of the stump inside the bladder. To achieve this result, it is essential that the surgeon combine the endoscopic supra-pubic pull-through with a manual “push through” action from the trans-perineal access, thereby avoiding any eventual injury to urethral tissue. Furthermore, we took advantage of the development of advanced operative instrumentation such as the flexible fiber-optic cystoscopy [20] and new suture materials.

A nodal point regarding this technique and the whole urethral surgery is the “ischemic fragility” of the urethral mucosa which is the main cause of perioperative complications [21]; as we are aware of this problem, we prefer to wait at least six months before implanting the AUS, thus avoiding ischemic injuries to the proximal part of the distal urethra.

Two patients presented urethral erosion after the second surgical step, so we removed the devices. As they are completely incontinent with a patent and stable lumen, we could advise re-implantation of the AUS, but we feel this indication should be limited to the irradiated patient. Adequate strategies such as adopting alternative cuff sites or performing trans-corporal cuff implantation allow us to avoid a second AUS revision [22] in this kind of patient.

We are aware that there is an important disadvantage in our two-stage approaches, which is the requirement of a second operation, but we believe that this is balanced by obtaining the recovery of the urethral tissue before implanting the sphincteric device, minimizing the risk of perioperative complications.

## 5. Conclusions

To treat patients that present urethral stricture or bladder neck contracture after prostatic surgery and failure of several endoscopic treatment, we advise performing a first-step surgery with a pure trans-perineal urethroplasty which is less invasive, easier to perform and has a lower operative time. If difficulties are encountered during the procedure, this may be switched to a combined trans-perineal/supra-pubic approach. After six months, when a stable patent urethral lumen is obtained, the patient can undergo AUS implantation.

## Figures and Tables

**Table 1:** Pre-operative patients features.

	Patient	Prostatic Surgery	Prior Urethrotomies	Prior TUR
Procedures with an end to end re-anastomosis	1	RRP	+	
	2	RRP	+	
	3	OSP		+
	4	OSP		+
	5	TURP		+
	6	RRP	+	

Procedures with the pull-through technique	7	LRP	+	
	8	RRP	+	
	9	RRP	+	
	10	RRP	+	
	11	LRP	+	
	12	RRP	+	
	13	RRP	+	
	14	LRP	+	
	15	RRP	+	
	16	RRP	+	
	17	RRP	+	

TUR: Transurethral Resection; RRP: Retropubic Radical Prostatectomy, LRP: Laparoscopic Radical Prostatectomy; OSP: Open Simple Prostatectomy; TURP: Transurethral Resection of Prostate.
